# Acute morbidity and complications of thigh compartment syndrome: A report of 26 cases

**DOI:** 10.1186/1754-9493-4-13

**Published:** 2010-08-19

**Authors:** Enes M Kanlic, Sarah E Pinski, Eric G Verwiebe, Jeremy Saller, Wade R Smith

**Affiliations:** 1 Department of Orthopaedic Surgery and Rehabilitation at TTUHSC in El Paso, Texas 4801 Alberta Ave., El Paso, Texas 79905, USA; 2 Department of Orthopaedic Surgery, University of Colorado School of Medicine, Aurora, Colorado, USA; 3 Department of Orthopaedic Surgery and Rehabilitation at TTUHSC in El Paso, Texas 4801 Alberta Ave., El Paso, Texas 79905, USA; 4 Department of Orthopaedic Surgery and Rehabilitation at TTUHSC in Lubbock, Texas, USA; 5 Department of Orthopaedic Surgery, Geisinger Clinic, 100N. Academy Ave, Danville, PA 17822, USA

## Abstract

**Background:**

To describe the patient population, etiology, and complications associated with thigh compartment syndrome (TCS). TCS is a rare condition, affecting less than 0.3% of trauma patients, caused by elevated pressure within a constrained fascial space which can result in tissue necrosis, fibrosis, and physical impairment in addition to other complications. Compartment releases performed after irreversible tissue ischemia has developed can lead to severe infection, amputation, and systemic complications including renal insufficiency and death.

**Methods:**

This study examines the course of treatment of 23 consecutive patients with 26 thigh compartment syndromes sustained during an eight-year period at two Level 1 trauma centers, each admitting more than 2,000 trauma patients yearly.

**Results:**

Patients developing TCS were young (average 35.4 years) and likely to have a vascular injury on presentation (57.7%). A tense and edematous thigh was the most consistent clinical exam finding leading to compartment release (69.5%). Average time from admission to the operating room was 18 +/- 4.3 hours and 8/23 (34.8%) were noted to have ischemic muscle changes at the time of release. Half of those patients (4/8) developed local complications requiring limb amputations.

**Conclusion:**

TCS is often associated with high energy trauma and is difficult to diagnose in uncooperative, obtunded and multiply injured patients. Vascular injuries are a common underlying cause and require prompt recognition and a multidisciplinary approach including the trauma and orthopaedic surgeons, intensive care team, vascular surgery and interventional radiology. Prompt recognition and treatment of TCS are paramount to avoid the catastrophic acute and long term morbidities.

## Background

Compartment syndrome of the thigh is a serious condition resulting from increased pressures within any of the three thigh fascial compartments. The most common etiologies include blunt trauma, with or without fracture, vascular injuries with ischemia reperfusion injuries, or frank bleeding into the myofascial spaces [1-3]. While the mechanism of compartment syndrome has been well described in the literature, the outcomes of those affected by thigh compartment syndrome have not. A review of the English literature reveals only two series, aside from isolated case reports, which document the outcomes of this condition. In some patients this syndrome leads to significant morbidity and mortality while in others complete recovery can be achieved with appropriate treatment. The disparity in outcomes may result from different mechanisms of injury, severity of soft tissue trauma, fracture, and/or the timing of treatment. Once thigh compartment syndrome is identified, immediate and complete compartment releases are required to prevent further ischemic insult to the tissues. Releases may not be indicated when the diagnosis is delayed more than twelve hours, as the complication rate increases precipitously. There are many reasons the diagnosis or intervention may be delayed including prolonged extrication, transfer time to the definitive treatment facility, or concomitant life threatening surgical or medical conditions. The obtunded and/or intubated patient, if the treating physician is not vigilant, is the most likely to experience a delay in diagnosis and subsequently the clinical outcomes for this group is poor. To further elucidate the timing of optimal intervention and better understand the impact of this injury on outcome, we reviewed the experience of two regional trauma centers.

## Methods

We performed a retrospective review of hospital charts at two level one trauma centers for those patients diagnosed with thigh compartment syndrome between December 1998 and December 2006. The Institutional Review Boards at both facilities approved the study protocol. Twenty-three patients with twenty-six thigh compartments syndromes were identified. The data collected included time and mechanism of injury, time to surgical decompression, associated injuries, vital signs and Glasgow Coma Score (GCS) on presentation, compartment pressure measurements, muscle appearance at time of surgery, subsequent surgical interventions, hospital length of stay, and ultimate outcome following definitive closure to include infections, nerve damage, chronic pain, and amputation.

In the awake and alert patient, the diagnosis of compartment syndrome was made most often using clinical criteria to include pain out of proportion injury, pain with passive stretch (complicated by the presence of fractures), palpation of compartment tension, and hypoesthesia or changes in motor function in the distribution of the nerves traversing the compartments in question (femoral, sciatic and obturator nerves) [4]. The presence or absence of distal pulses was noted but not used as a sole criterion for compartment release as several patients had vascular injuries below the level of Hunters canal. The changes in distal pulses may result from a late compartment syndrome or an acute vascular injury. For non-cooperative, obtunded and polytrauma patients intubated prior to examination, both compartment pressure measurements and clinical exam finding of palpably tense compartments were used in the decision making process. Absolute compartment pressures greater than thirty millimeters of mercury were considered diagnostic, especially in the critically ill patient where blood pressure fluctuations may alter tissue perfusion pressures acutely [5]. In those patients not at risk for development of hemorrhagic shock, a delta pressure (Δp) of less than 30 mm Hg was used as an indication for fasciotomies [6]. The compartments of the thigh were released through a single, long, lateral incision to access the anterior compartment directly and posterior compartment through the lateral intermuscular septum [7]. After these compartments were released, a repeat evaluation of the medial (adductor) compartment was performed. If pressures remained elevated, the medial compartment was released through a separate incision [8]. Assessment of muscle viability was made at the time of surgical decompression using the bovie for electrical stimulation in conjunction with contractility when grasping muscle tissue with forceps. If muscle twitch was not noted following stimulation with electro-cautery, then appropriate debridement was carried out until bleeding and contractile muscle was encountered. Most incisions following fasciotomy were left open and treated with either non-adhesive dressings or a vacuum assisted device. Split-thickness skin grafting or delayed primary closure was performed after subsidence of swelling, usually in time frame of five to seven days. If necrotic muscle was encountered, a thorough debridement was carried out, a drain placed, and primary closure easily performed (without tension) to prevent contamination or infection. In our cohort, infection was determined clinically based on inspection of the wound and surrounding soft tissues. Those patients defined as having fasciotomy site infections developed marginal erythema, increased wound drainage, and/or wound dehiscence often necessitating a return to the operating room for irrigation and debridement during their initial hospitalization. Complications regarding nerve injury were documented if there was a significant decrease in muscular strength as compared to the contralateral extremity of at least two grades.

## Results

Twenty-three patients with twenty-six thigh compartments syndromes were identified. The average patient age was 35.4 years (range of seventeen to sixty-four years). The primary mechanism of injury in these patients was motor vehicle collision in 11/23 (48%), blunt injuries to the thigh 9/23 (39.1%), isolated gunshot wound to the thigh 2/23 (8.7%), and intramuscular injection of drugs 1/23 (4.3%). Bilateral thigh compartment syndrome was noted in 3/23 (13.0%).

**Figure 1:**
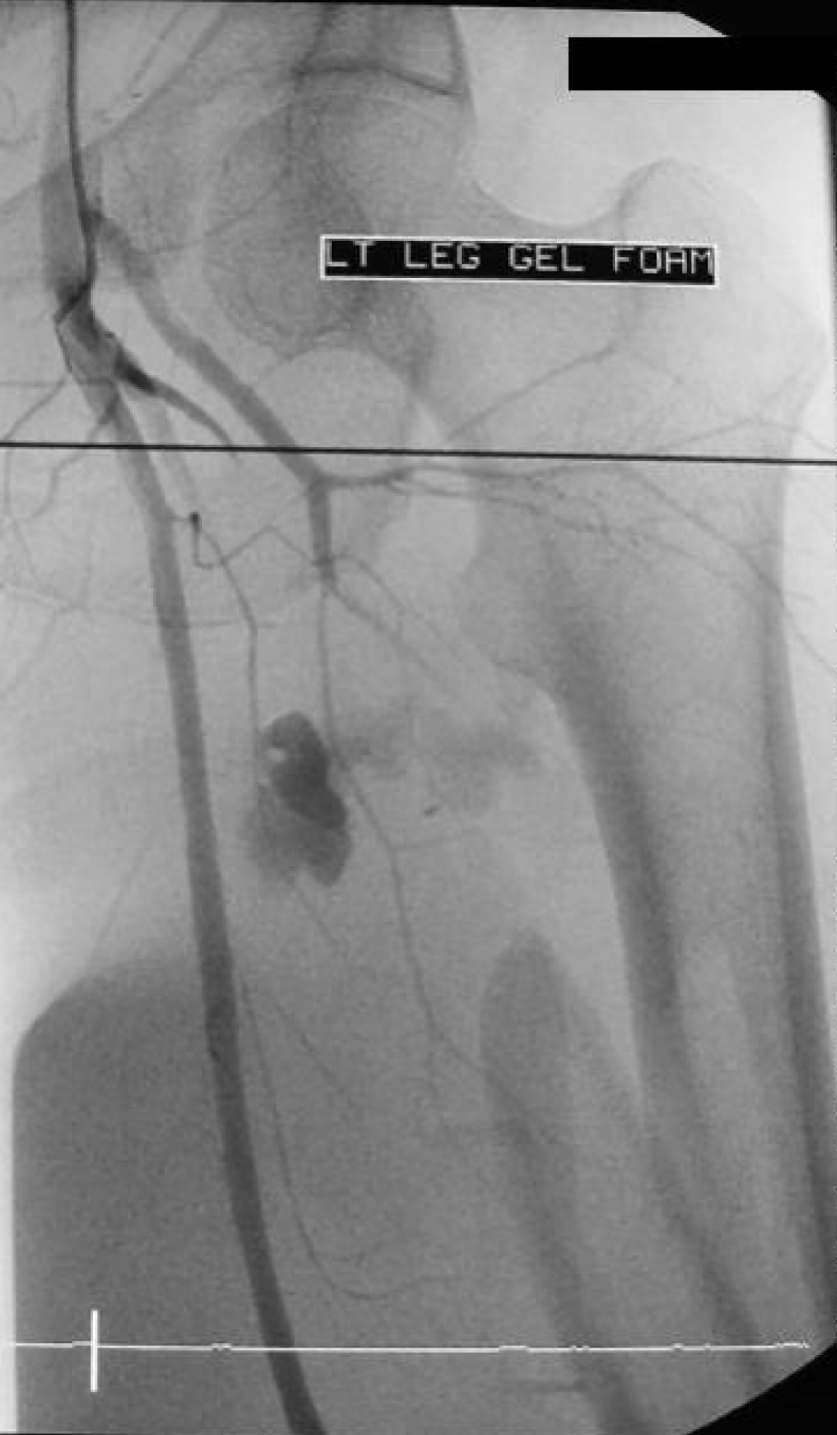
**Angiogram of thigh vessels in young female patient on bicycle who was hit by car one hour before admission**. She was in hemorrhagic shock, with peripheral pulses and sensation present, with unstable femoral shaft fracture and with clinical presentation of thigh compartment syndrome. Arterial bleed was diagnosed by angiography and damaged arterial brunch was promptly embolized. All three thigh compartments were released, femur fracture was fixed with intramedullary nail and patient had good final outcome.

A vascular injury which may have contributed to development of thigh compartment syndrome was present in 15/26 (57.7%) cases (Figure 1). Few of vessels injuries were at multiple levels, but most commonly were involved superificial femoral artery (six cases), iliac artery (three), popliteal artery (two), and the rest are single injuries (superficial gluteal artery, profunda femoral artery, inferior vena cava and popliteal vain) (Table 1). Diagnosis of thigh compartment syndrome with vascular compromise was made clinically in all patients, and just in two cases pressures were measured as well. Only 2/15 (13%) patients with associated vascular injuries did not require release of leg compartments. Looking at the whole cohort of 26 thigh compartments only 8 (30.8%) had just thigh compartments release, rest of them had leg releases as well. 11/16 patients with vascular compromise and with avaliable data, had fasciotomies done at the same time when vessel injury was addresses. 3/16 had releases done next day, and 2/16 two days after original surgery.

**Table 1:** Mechanism producing thigh compartment syndrome

Blunt trauma	73% (19/26)
Penetrating trauma	11% (3/26)

Vascular injuries	57.7% (15/26)

Compartment pressures were measured in 10/23 patients (43.5%) with the average compartment pressure measurement being 57+/- 6 mmHg. In general, the pressures in the anterior compartment were highest.

**Figure 2:**
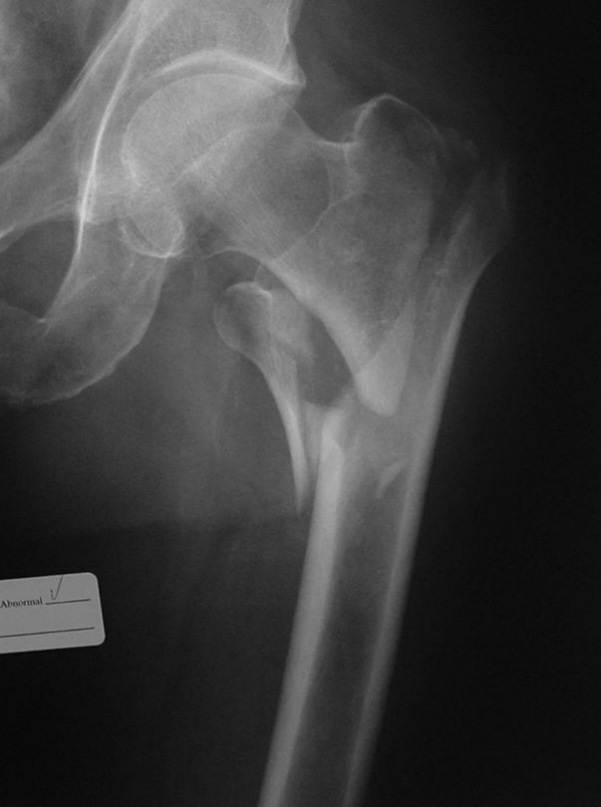
**Antero-posterior x-ray of unstable pertrochanteric fracture sustained after simple fall by an elderly patient**. Eighteen hours after the injury he started developing signs of acute thigh compartment syndrome and was taken urgently to operating room for anterior thigh compartment fasciotomy. Muscles were still valuable and fracture fixation with cephalomedullary nail was done immediately after. Patient did not have any history of hemorrhagic diathesis, was not taking any blood thinners and there were no abnormalities on patient routine preoperative workup (PT, PTT, INR, platelets). He recuperated well.

Average time from admission to compartment releases in the operating room was18 +/- 4.3 hours. All patients with compartments release time longer than 12 hours (34.5 hours on average) were uncooperative, obtunded and multiply injured patients (7/23). Nonviable muscle was noted in 8/23 patients (34.8%) and follow-up information was available on 5/8 patients. Of these, 4 patients required amputation with 2 of those being above knee (Table 2). Four of 23 patients (17.4%) died resulting from multisystem trauma and high energy mechanisms of injury as often seen with thigh compartment syndrome. Ipsilateral femur fractures were noted in 8/19 patients (42%; Figures 1,2).

**Table 2:** TCS Complications

Mortality	17.4% (4/23)
Muscle necrosis	34.8% (8/23)

Amputation	17.4% (4/23)

Tense compartments were noted in 16/23 patients (69.5%), diminished pulses to the lower extremities in 8/23 (34.8%), pain with passive stretch in 6/23 (26.1%), and paresthesias in 5/23 (21.7%), of patients. Prophylactic compartment releases were carried out in 2/23 (8.7%) patients due to ipsilateral vascular repair.

Wound closure data was available on 23/26 thighs. Fasciotomies wounds closure was an average 6.5 days. Primary closure of the fasciotomy sites was performed in 3/26 (13%) thighs, delayed primary closure in 11/26 (48%), and split thickness skin grafting in 9/26 (39.1%). The two patients requiring an above knee amputation received split thickness skin grafting.

## Discussion

Thigh compartment syndrome remains a rare clinical entity with only two complete series reported in the English literature comprised of 45 patients [8,9]. The variability in patient outcomes following treatment of TCS is not only a function of injury mechanism but in the timely and accurate diagnosis of reversible muscular ischemia and immediate surgical intervention. In a canine model, Matava et al. showed that *eight hours *of increased intracompartmental pressures to within 20 mm Hg of the diastolic blood pressure (Δp) was the critical threshold for ischemic muscle necrosis [10]. In an earlier study, Heppenstall et al. also showed that ischemic changes may be present in a *four to six hour *time frame when the Δp approached 20 mm Hg. They also suggest that periods of *hypotension *may result in muscle damage at even lower compartment pressures [11]. These studies highlight the need for increased emphasis to be placed on compartment pressure monitoring or serial examinations in those patients who are at risk for developing TCS as the window for successful treatment may be very narrow. The side port needle used with the "Stryker intra-compartmental pressure monitor system" (Stryker^® ^Instruments, Kalamazoo, MI), remains a mainstay in the measurement of compartment pressures at our institution. Though it provides only a single data point regarding a condition that is both continuous and dynamic, its accuracy and simplicity often help to confirm compartment syndrome in patients with a confusing exam or in those who are unresponsive. Continuous monitoring of extremities at risk using an arterial line manometer as described by Matsen et al. may be a consideration as well as this gives a more comprehensive set of data [5]. Newer technologies include infrared imaging of extremities in the trauma setting, using temperature differences between the proximal and distal skin surfaces in order to make the diagnosis [12]. This technology is promising though requires more equipment in the emergency room setting, software, and personnel for data interpretation.

Whereas fracture is the leading cause of compartment syndrome in the leg, thigh compartment syndrome is more commonly associated with blunt trauma or vascular injury [8,9,13]. Hope et al. reported on 151 cases of acute compartment syndromes, including both upper and lower extremities, noting that only 40% of those developing compartment syndromes of thigh could be attributed to a fracture. This contrasts sharply with the 77.8% of his patients whose compartment syndromes of the leg were attributed to tibia fractures. In addition, compartment syndrome of the leg was diagnosed in 59.6% of this cohort while thigh compartment syndrome was present in only 6.6% [14]. This makes selection of those who are at risk for developing TCS more difficult as the sentinel event may be more obscure than a displaced femoral shaft fracture and the frequency at which this is seen is considerably lower than that of the leg. Case reports of thigh compartment syndrome highlight the diverse mechanisms of injury to include exercise induced [15-18], quadriceps tendon rupture[1], drug popping [19], crush injury [2], thigh contusion [20-23], aggressive resuscitation in the trauma setting [24], positional ischemia [3,25], aneurysm [26], following joint replacement [27], deep venous thrombosis [28], vascular injury [29], and of course fracture [7].

In our study, the vast majority of TCS resulted from blunt trauma to the pelvis or lower extremities in 19/26 (73%) thighs. Of these patients, 8/19 (42%) had fractures of the ipsilateral femur (Figures 1,2). This compares with Mithöfer et al. who reported a similar mechanism in 85% of their patients and ipsilateral femur fractures in 15/29 compartment syndromes (52%) [9]. Schwartz et al. reported a similar mechanism in 76% of their 17 patients with 58% having ipsilateral femur fractures [8]. The next most common mechanism in our cohort was penetrating trauma and gunshot wounds resulting in 3/26 compartment syndromes (11%). Vascular injuries were present in the ipsilateral extremity in 15/26 cases, making this diagnosis likely in the face of a developing TCS, though only 10/26 involved the superficial femoral, iliac, or profunda femoral vessels. This rate of vascular injuries is higher than that previously published by Mithöfer et al. 4/28 patients (14%) and Schwartz et al. with 4/17 patients (23.5%) [8,9].

The most consistent objective exam finding leading to diagnosis of TCS was a tense and edematous thigh noted in 18/26 compartment syndromes. While pain and paresthesias to the effected extremity have been well supported in the literature as an indication for impending compartment syndrome, this was documented in only 8/26 thighs lending to the high energy mechanisms and multiple system involvement in these patients who are not able to cooperate.

The difficulty in managing patients who are at risk for developing TCS is early recognition, especially in the poly-trauma patient who is intubated and sedated. While TCS may be obvious on initial exam in the trauma bay, it also may develop insidiously over the next 24-48 hours as seen in two patients in our series. It is critical to identify at what time intracompartmental pressures have reached the critical, tissue "suffocating" level. Our average arrival to the operating room for compartment releases was 18 +/- 4.3 hours from the time of admission to our facility. At surgical decompression 35% of our patients had nonviable muscle noted and half of those patients required amputations. Special consideration needs to be given to those patients known to have greater than 12 hours of ischemia time. This time frame of twelve hours is a relative estimate, as it is hard to know at what point following injury that the intra-compartmental pressures have reached ischemic levels. Repeated examinations (including intra-compartmental pressures monitoring) are possible when patient arrives early after injury to the hospital. Decision making is easier and majority of those compartments will be opened. When timing of injury is known, and when arrival to the hospital is twelve or more hours after, it is better not to open ischemic muscles. Risk of infection and amputation is probable, and patient should be treated non-operatively, medically (rapid fluid resuscitation) in intensive care setting with general idea to manage rhabdomyolysis and avoid acute renal failure.

**Figure 3:**
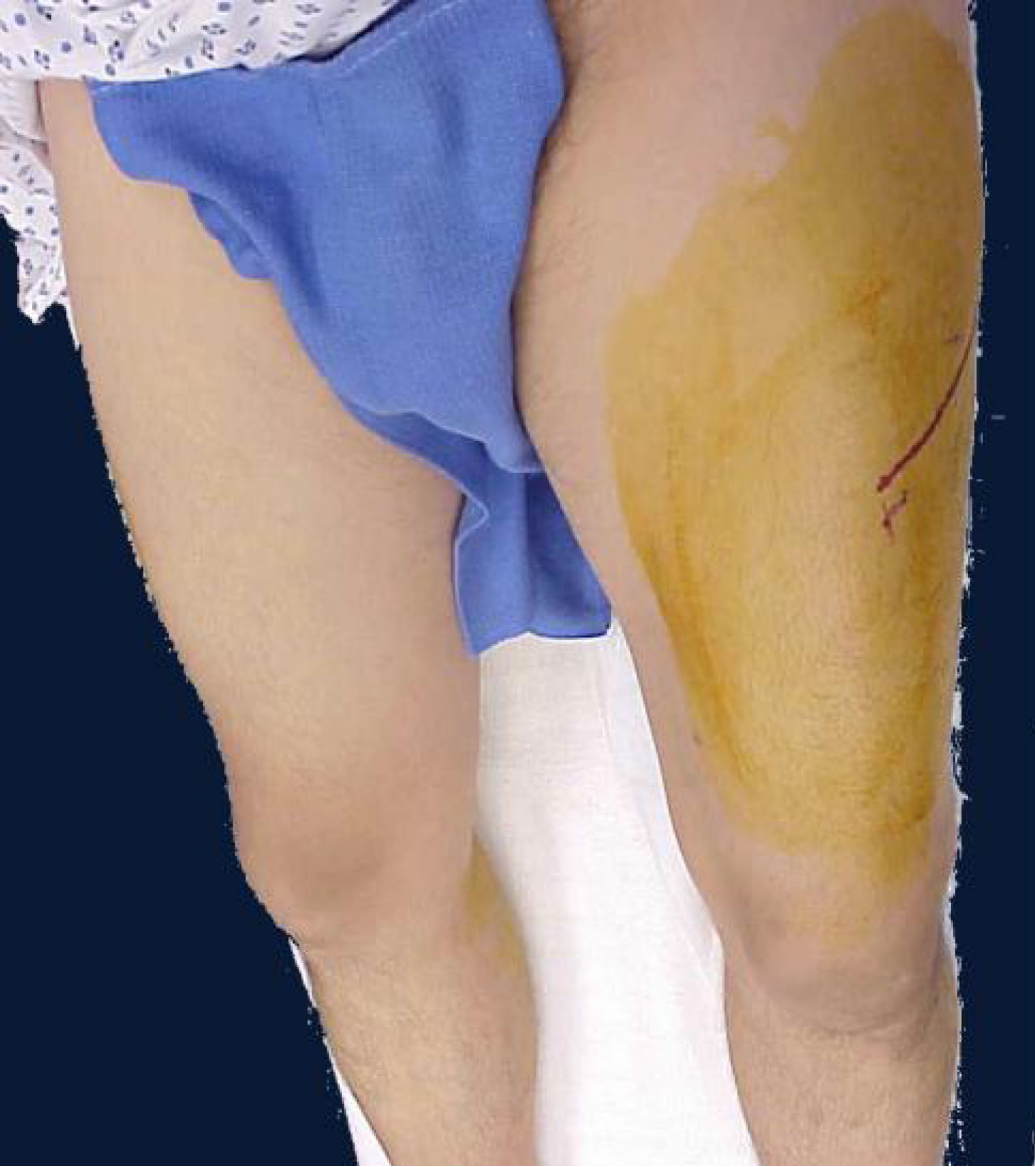
**Clinical photo of male patient in his forties who was hit by car day before**. He presented with significant swelling of his left thigh and gradually increasing pain especially on attempts of knee flexion. Anterior thigh compartment was very tense, measured pressure was close to diastolic pressure; distal pulses and sensation were intact.

**Figure 4:**
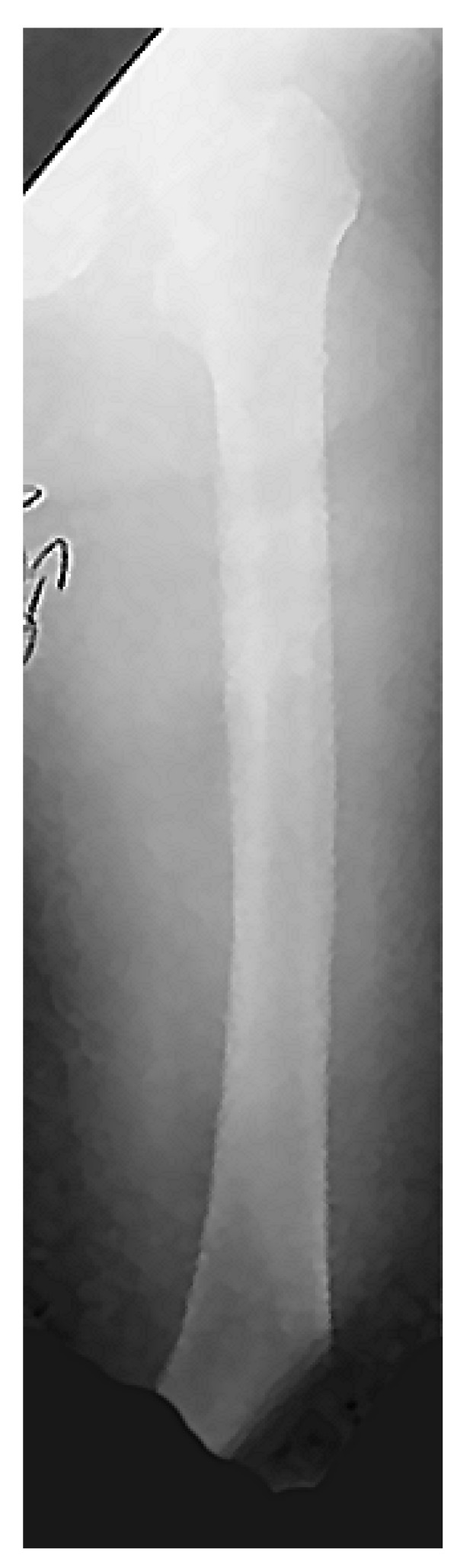
**Antero-posterior femur x-ray has not revealed any fracture**.

**Figure 5:**
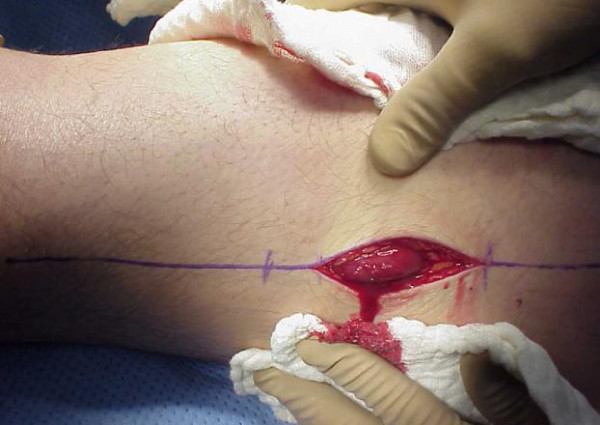
**Small incision thorough the fascia allowed for inspection of quadriceps muscles viability**. Muscle was alive reacting promptly with contractions on pinch with forceps and electro-cautery touch.

**Figure 6:**
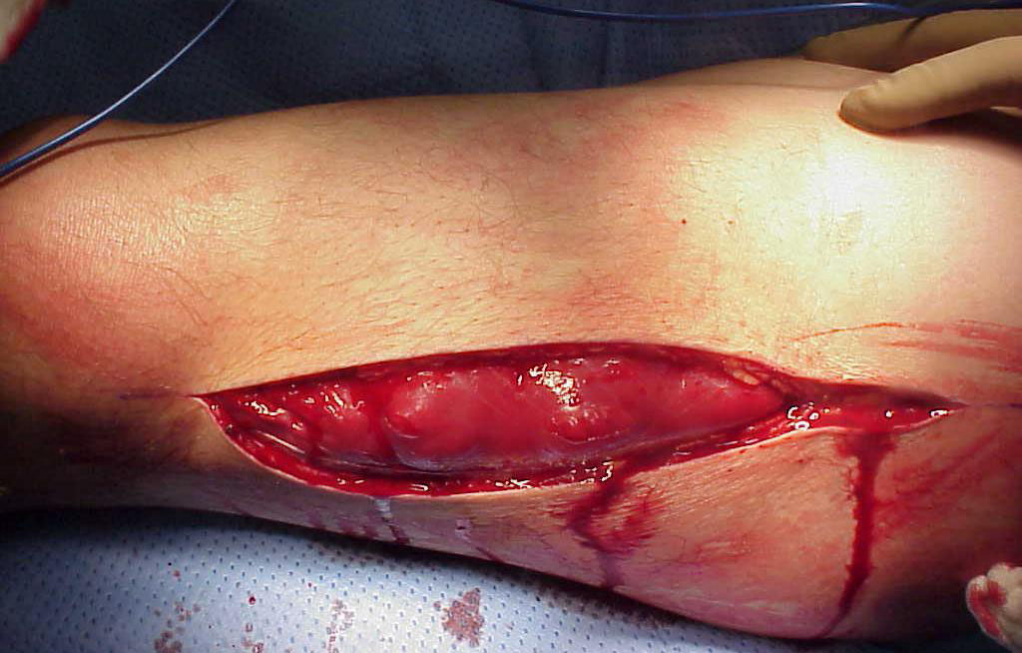
**Partial fasciotomy with muscle still under pressure**.

**Figure 7:**
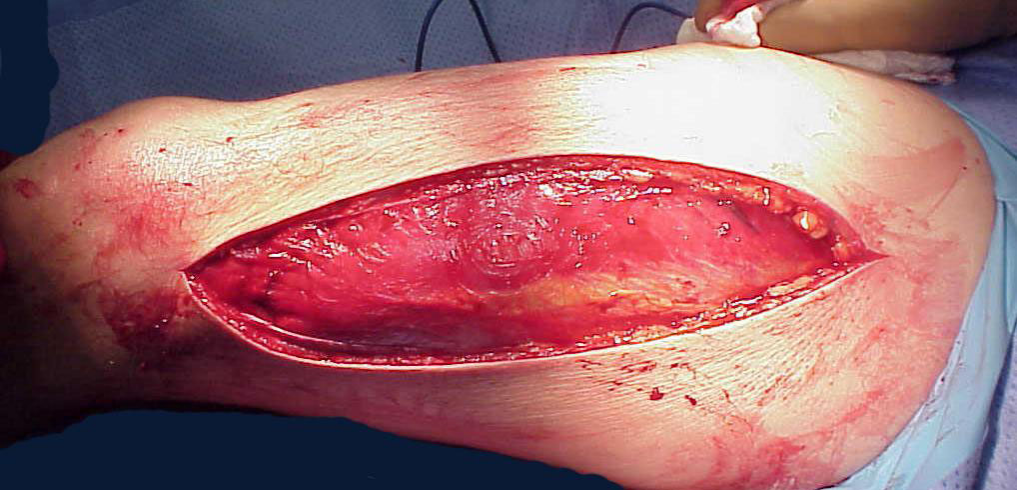
**Complete antero-lateral fasciotomy was done with muscle escaping high intra-compartmental pressure**.

**Figure 8:**
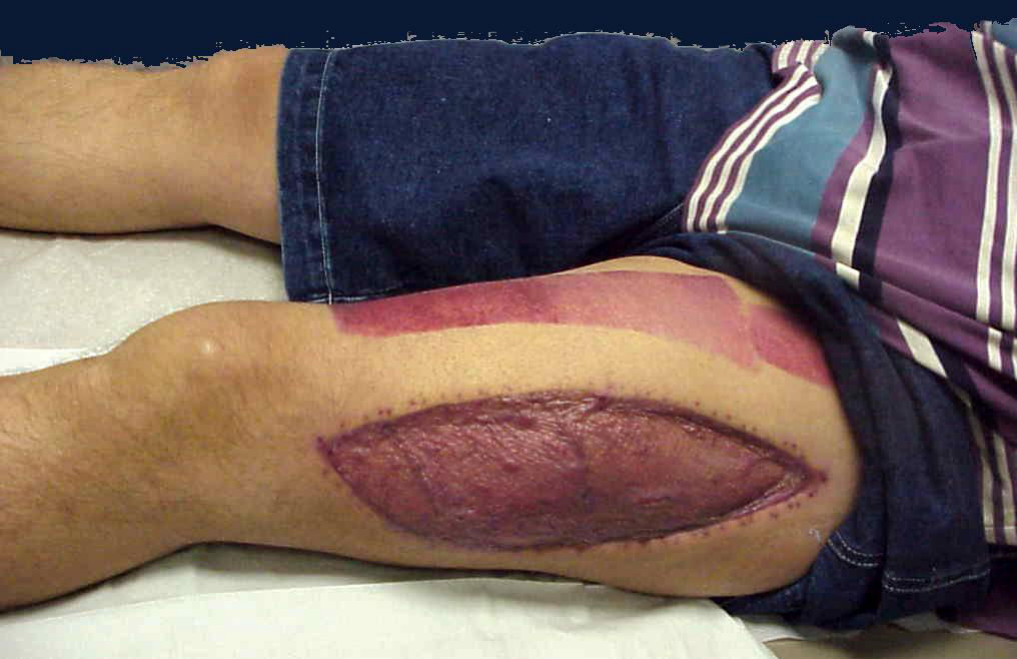
**Clinical photo of well functioning patient few weeks after the injury**. The soft tissue defect was treated with negative pressure wound therapy immediately after fasciotomy followed by successful split thickness skin grafting six days after.

Similar approach we have to take in cases after crush injuries (often seen in earthquake). Crush syndrome is different than acute compartment syndrome; compartments are in general less tense on examination, less painful, sensation deficit is patchy (not in distribution of specific nerve) and if opened, muscles will be bleeding although they are not valuable and will easily get infected. In situation of clinical compartment syndrome, when we are not sure whether is too late to intervene, small incision in operating room could be done and muscles viability checked (Figure 3). If muscles are reacting on stimulation, compartment should be released (Figures 4,5,6,7,8), if not - closed and allow for tissues scarring to happen in attempt to avoid infection and amputation. Sheridan et al. showed that when the fasciotomy is performed more than twelve hours after diagnosis, complication rate increased from 4.5% to 54% with 1 in 5 patients requiring amputation [30]. Mithöfer et al. reported similar times of 11.1 +/- 3 hours for all patients and 14.5 +/- 5.8 hours in those patients presenting without fractures, though they reported a much lower complication rate of 18% [9]. Schwartz et al. did not report the time to fasciotomy from injury but from the time it was diagnosed and averaged 4 hours. They reported a much higher wound complication rate of 66% though there were no reports of amputations [8].

Definitive treatment of the fasciotomy sites often (39% of patients) required skin grafting for closure which is comparable to the Schwartz et al. cohort (41%) and significantly higher than the Mithöfer cohort (12%). Our mortality rate of 17.4% highlights the severity of injuries sustained by this cohort. Previous series have shown mortality rates to be between 11% - 47% depending on the study [8,9].

## Conclusion

Traumatized limbs with vascular compromise (diminished or absent peripheral pulses) have high risk of developing thigh (57.7% in our study) and leg compartment syndrome. A careful clinical exam at the time of admission and diligence with serial examinations of the extremity at risk may identify the majority of TCS in the awake and alert patient. Objective measures need to be employed in the obtunded or multiply injured patient in the form of single or continuous intra-compartmental pressure monitoring. The key to treatment of pending or acute muscle and nerve ischemia, e.g. compartment syndrome in any location remains prompt diagnosis and expedient compartment releases. For patients where the diagnosis has been delayed for more than twelve hours (or even 6 hours in the face of high intracompartmental pressures), strong consideration should be given to avoid exposing damaged (necrotic) tissues to the environment due to increased infection risk and probable amputation. Aggressive management of medical issues to prevent renal damage may better serve these patients, allowing for preservation of limb, life, and late reconstructions [31,32].

In the subset of patients, when we are not certain about condition of intracompartmental tissues (lasting of increased pressures), a small incision to allow access for testing of muscle viability should be considered. If the long fasciotomy incisions have already been made, necrotic tissues must be removed and the wound should be closed in an attempt to prevent infection of remaining tissues.

## Competing interests

The authors declare that they have no competing interests.

## Authors' contributions

EMK and WRS were responsible for developing the idea of this study and the study design as well as being directly involved in the treatment of the majority the patients involved. They were also involved in reviewing and editing the manuscript. EGV performed the literature review and drafting of the manuscript as well as evaluating all data collected. JS and SEP were involved in the records review and data acquisition for all patients at both facilities. All authors read and have approved the final manuscript.
